# T-DNA-genome junctions form early after infection and are influenced by the chromatin state of the host genome

**DOI:** 10.1371/journal.pgen.1006875

**Published:** 2017-07-24

**Authors:** Shay Shilo, Pooja Tripathi, Cathy Melamed-Bessudo, Oren Tzfadia, Theodore R. Muth, Avraham A. Levy

**Affiliations:** 1 Department of Plant and Environmental Sciences, Weizmann Institute of Science, Rehovot, Israel; 2 Department of Plant Pathology, Volcani Center-ARO, Bet-Dagan, Israel; 3 Department of Plant Systems Biology, VIB, Technologiepark 927, Ghent, Belgium; 4 Department of Plant Biotechnology and Bioinformatics, Ghent University, Technologiepark 927, Ghent, Belgium; 5 Bioinformatics Institute Ghent, Ghent University, Technologiepark 927, Ghent, Belgium; 6 CUNY Brooklyn College, Department of Biology, Brooklyn, NY, United States of America; The University of North Carolina at Chapel Hill, UNITED STATES

## Abstract

*Agrobacterium tumefaciens* mediated T-DNA integration is a common tool for plant genome manipulation. However, there is controversy regarding whether T-DNA integration is biased towards genes or randomly distributed throughout the genome. In order to address this question, we performed high-throughput mapping of T-DNA-genome junctions obtained in the absence of selection at several time points after infection. T-DNA-genome junctions were detected as early as 6 hours post-infection. T-DNA distribution was apparently uniform throughout the chromosomes, yet local biases toward AT-rich motifs and T-DNA border sequence micro-homology were detected. Analysis of the epigenetic landscape of previously isolated sites of T-DNA integration in Kanamycin-selected transgenic plants showed an association with extremely low methylation and nucleosome occupancy. Conversely, non-selected junctions from this study showed no correlation with methylation and had chromatin marks, such as high nucleosome occupancy and high H3K27me3, that correspond to three-dimensional-interacting heterochromatin islands embedded within euchromatin. Such structures may play a role in capturing and silencing invading T-DNA.

## Author summary

*Agrobacterium tumefaciens* mediated T-DNA integration is an important tool for genetic engineering in plants. This work compares the genetic and epigenetic landscapes of T-DNA-genome junctions under selective and non-selective conditions. Under selection, preferential junctions in low-nucleosome occupancy and hypomethylated regions were found. In the absence of selection, these biases disappeared and T-DNA-genome junctions were uniformly distributed with a preference for 3D-interacting heterochromatin islands embedded within euchromatin, suggesting that many integration events become transcriptionally inactive.

## Introduction

*Agrobacterium tumefaciens* is the causative agent of crown gall disease [1–3], however, disarmed strains of *A*. *tumefaciens* are widely used to create genetically modified plants. *A*. *tumefaciens* transfers a single stranded T-DNA molecule into the plant host cell together with other virulence proteins [1–3]. The single stranded T-DNA forms a complex with a single VirD2 protein covalently bound to its 5′ end and with several VirE2 proteins bound along the single-strand DNA. This complex is transported to the nucleus where the T-DNA integration process takes place. The T-DNA-genome junctions at the 5′ end are much more precise than at the 3′ end [4,5], likely owing to the role of VirD2 in protecting the 5′ end [6]. By contrast, the frequent occurrence of DNA structural variations at the genome-T-DNA 3′ end junctions were recently shown to be due to the error-prone activity of the plant polymerase theta, a protein essential for T-DNA integration [7]. Several lines of evidence showing that the T-DNA integrates at induced DNA double stranded breaks (DSBs) together with the typical non-homologous end-joining (NHEJ) footprints supports a model of T-DNA integration via a DSB repair pathway [8,9]. However, integration via a double or single stranded T-DNA intermediate remains possible [10–12].

Several questions remain with regards to T-DNA integration: for example, the timing of integration following infection, and whether there are preferences (genetic and epigenetic) for T-DNA integration, are not fully understood [2,13,14]. The distribution of T-DNA integrations in the *Arabidopsis* genome has been examined previously, with the reports arriving at conflicting conclusions. First, a study examining over 80,000 independent integration events showed a bias for T-DNA integrations in gene-rich areas [13]. However, these results used selective conditions and may not have been able to detect T-DNA insertions into transcriptionally inactive regions. More recent studies based on the analysis of events obtained under non-selective conditions concluded that the location of T-DNA integration events is essentially random [14,15]. However, the relatively low number of T-DNA integration events analyzed under non-selective conditions limits the ability to identify biases in integration that may exist. These findings raise the need for an unbiased, high-throughput, system that identifies T-DNA-genome junctions and incorporates recent epigenetic data [16–19]. The epigenetic landscape is known to be involved in processes that are relevant for T-DNA integration, such as silencing of expression by H3K27me3 [20] or DNA methylation [17], DNA recombination [21–23], the formation of specific chromosomal domains [19], and the 3D organization of the DNA in the nucleus [24–26].

In an effort to gain an unbiased perspective of T-DNA integration, we modified the adapter-ligation mediated PCR method [27] from a selection based method to a selection-free method similarly to what was done recently for mapping of HIV integrations in human [28,29]. DNA was extracted from *Agrobacterium*-infected roots at several post-infection time points and T- DNA to genomic DNA junctions were amplified, sequenced and mapped to the *Arabidopsis* genome without the need to grow a transformed plant. Our data indicate that T-DNAs can form junctions with the genome relatively quickly (within 6 hours). Furthermore, our results show that in the absence of selection T-DNA junctions form throughout the genome with enrichment in regions of high nucleosome occupancy, while under selective conditions junctions were preferentially in hypomethylated regions with low nucleosome occupancy. In general, T-DNA junctions have some bias at the sequence level, with preferential formation in regions that share microhomology to the T-DNA borders and with some enrichment in AT-rich regions. In summary, our analysis shows that unselected T-DNA-genome junctions, in comparison to junctions formed under selective conditions, are distributed more uniformly, but not randomly, across the genome with preferences for AT-rich sequence motifs and for H3K27me3-enriched heterochromatin regions embedded in euchromatin.

## Results

### High-throughput detection of unselected T-DNA-genome junctions

**Fig 1:**
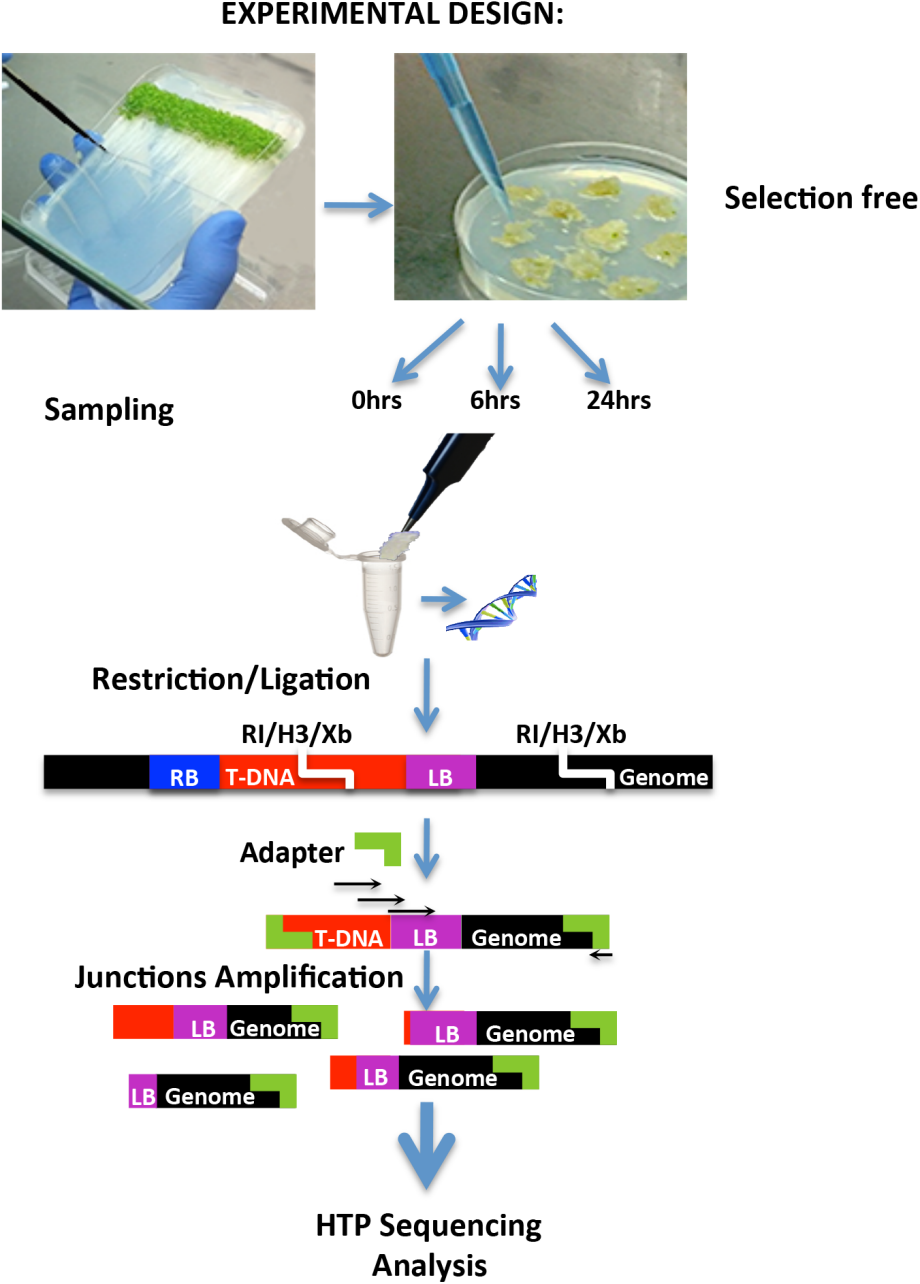
Experimental design–*Arabidopsis* roots were infected with *A*. *tumefaciens*. DNA was extracted at 0, 6 and 24 hours post infection. Extracted DNA was digested with 3 restriction enzymes: *Eco*RI (RI), *Hind*III (H3) and *Xba*I (Xb). An adapter was ligated to the overhang end of the digested DNA. T-DNA-genomic junctions were amplified using three different primers from within the T-DNA and one primer from the adapter (primers—black arrows, LB–left border, RB–right border). Amplicons were sequenced using high throughput sequencing. Adapter to adapter products were reduced as detailed in O’Malley et al. 2007 [27].

In order to systematically characterize T-DNA-genome junction sites in the genome without the use of selection, roots of young *Arabidopsis* seedlings were cut, infected with *A*. *tumefaciens*, and their DNA was extracted (see Fig 1 and details in Methods). We combined the previously described adapter ligation-mediated PCR method [13,27] for T-DNA junction detection with high-throughput sequencing. Due to the expected presence of deletions during T-DNA-genome junction formation, we used three primers located -11, -30 and -70 bp from the left border terminus. These junctions likely correspond to T-DNA integration events, however, because we did not characterize both borders of the T-DNA, it is possible that some of these junctions could be from T-DNA integration intermediates.

The high-throughput sequencing data was filtered to ensure that only high quality reads with minimal possible artifacts were used in the analysis. Only reads with a Phred score above 25 for every base were included. The PCR product from a given junction could be represented by multiple reads depending on the depth of sequencing and variations in amplification efficiency from junction to junction. In keeping with this, we found in our analysis of the sequence data that 99% of the junctions were represented by multiple sequence reads. The multiple reads from a single junction were collapsed to single events after alignment. In order to avoid false positive results we used a control infection where DNA was extracted immediately after the inoculation with *A*. *tumefaciens* at time zero (T0) leaving no opportunity for T-DNA integration to occur. DNA from the T0 time point went through the entire protocol of T-DNA junction detection along with all other samples.

As expected, the number of reads obtained from the T0 control prior to any filtering was only 2.0–3.5% the number of reads from DNA extracted 6 and 24 (T6 and T24) hours post infection (S1 Fig). All reads from T6 and T24 that matched reads from T0 were removed from the analysis. After alignment of the T0 reads to the reference genome we found that some genomic locations showed preferences for artifacts, namely if the number of reads mapped to a given location was above the mean plus two times the standard deviation. In these cases the T0 sites representing likely artifacts were masked in the downstream analysis. Another possible cause for false positive detection of a junction is the result of a partial match between the primer and the genome. In order to remove artifacts that may have resulted from amplification from the genome in the absence of junction formation, we required that every read map to both the genome with at least 22 bp perfect match and to the T-DNA sequence with a match to the primer plus four nucleotides from the left border (the inclusion of the four additional left border nucleotides beyond the primer sequence is to reduce the chances of including any events resulting from 3’ primer invasion into the host genome). Most of the reads (~90%) mapped unambiguously to a unique target location over the genome. The location of the remaining ~10% reads was chosen according to the best bit score given by blast. Finally, our system was based on amplification with three different primers from within the T-DNA. In order to avoid counting the same read more than once and to discard all the PCR duplicates, reads were collapsed so that those from the same primer with junctions mapping 10bp apart were counted only once. In total, we identified 2801 junctions; 1899 of these in T6 and 902 in T24.

### Distribution of selected versus unselected T-DNA-genome junctions along chromosomes

**Fig 2:**
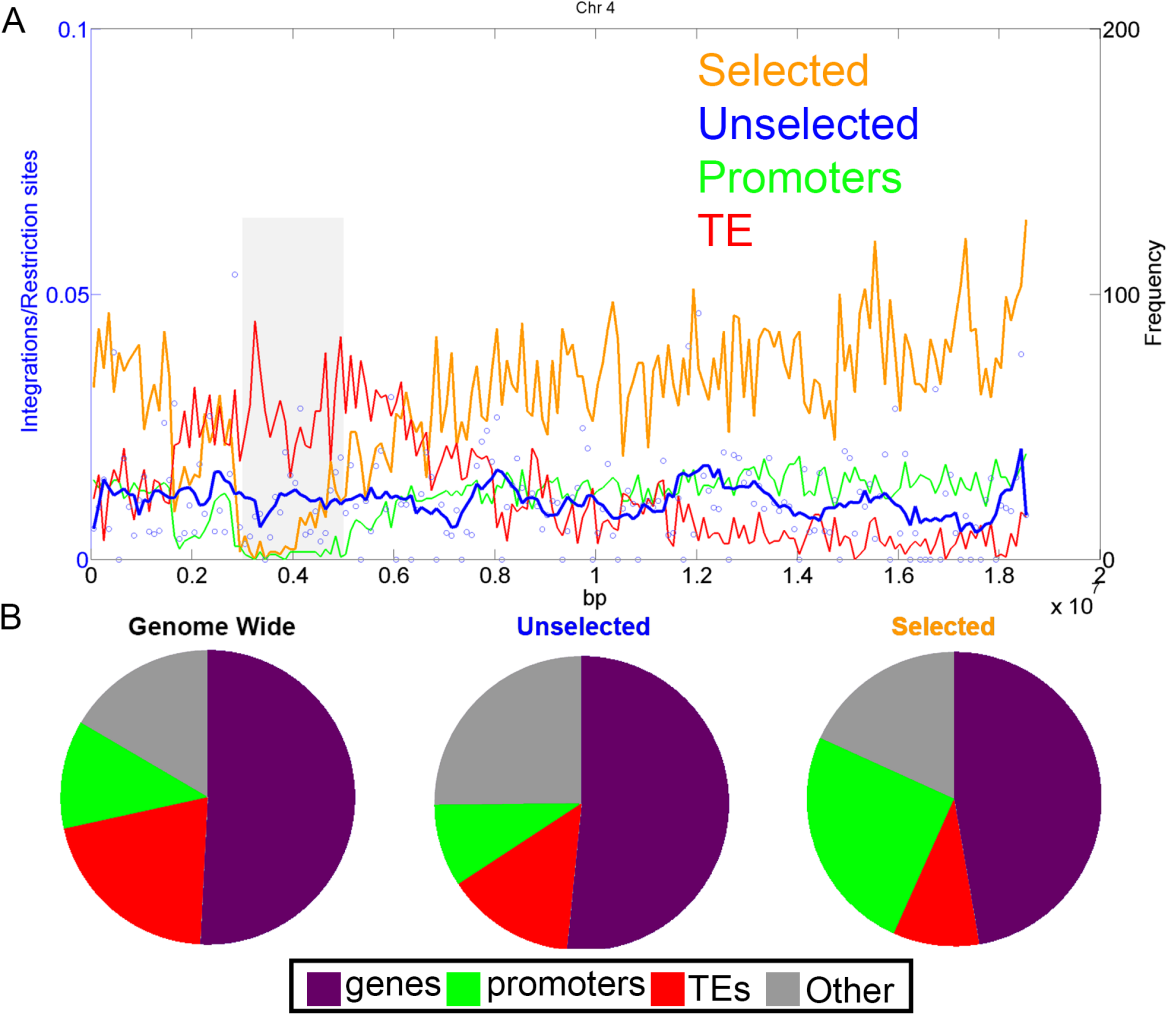
Association of genomic features with T-DNA-genome junctions under selective and non-selective conditions. A–The genomic distribution of unselected and selected T-DNA–genome junctions across chromosome 4. The numbers of T-DNA–genome junctions (circles, and smoothed blue line) do not show a correlation with the distribution of transposons (TE, red line) and promoters (green line). T-DNA integrations under selective conditions (orange line) correlates with genes/promoters [13]. B- The portion of each genomic feature: TE (red), genes (purple), promoters (green) and the remaining regions (other, grey). The portion of genomic features is represented across all the genome (genome wide) and according to the number of T-DNA–genome junctions: without selection (Unselected) and with selection (Selected- data from Alonso et al., 2003 [13]). Selected events [13] show an enrichment in promoters (χ^2^ test, compared to unselected events, p = 2.88E-24) and a decrease in TE regions (χ2 test, compared to unselected events, p = 0.005).

From the 2801 unselected T-DNA-genome junctions that we identified, the distribution of these junctions in chromosome four is shown in Fig 2 (blue line and circles). There is no obvious integration bias when considering the main chromosomal domains (centromeric, pericentric, distal, subtelomeric or telomeric). Similar results were found for the rest of the genome (S2 Fig). By contrast, the analysis of kanamycin-selected T-DNA integrations (Fig 2 orange line) suggests that T-DNAs tend to integrate into gene rich, transposon poor, regions, with low frequency of insertion in pericentric and centromeric regions [13]. In detail, integrations into pericentromeric regions, corresponding to 8.3% of the genome, were rare in the selection-based data, namely 2% in reanalysis of these data. In the unselected data the ratio of T-DNA-genome junctions in pericentromeric regions was more than four times higher (8.2% of the events) and close to the percent of pericentromeric regions in the genome, consistent with the expected value for random integration.

Since the original publication of Alonso et al. (2003) [13] there has been much improvement in the genome annotation as well as with epigenetic data. Therefore, we reanalyzed the ~80,000 selected integration events with updated genome annotation, TAIR10, at a single base resolution, characterizing the genomic features at the site of the integration. Alonso et al. (2003) proposed that integrations occur in genic regions [13] which we confirmed (Fig 2A). In addition, we showed that integrations under selective conditions are biased toward promoters (green line, Fig 2B). By contrast, the unselected data did not show a bias in junction formation in genes, promoters or transposable elements (TE, red line, Fig 2A and 2B)

### Sequence bias at sites of unselected T-DNA-genome junctions

We did not find any bias for or against the GC content in single, di, or tri nucleotides. This confirms and extends the data from Kim et al. 2007, who reported only minor bias of GC content at the T-DNA-genome junctions of unselected events [14]. To further investigate the possible effect of the host genome sequence on the localization of junctions, we used two algorithms for sequence motif analysis, HOMER [30] and MEME [31,32]. The genome was divided into non-overlapping bins of 400bp and bins containing at least one event were used for the analysis while random sampling of the genome was used as a control. Overall, 2328 sequences of 400bp length each were used as a dataset for sequence investigation.

**Fig 3:**
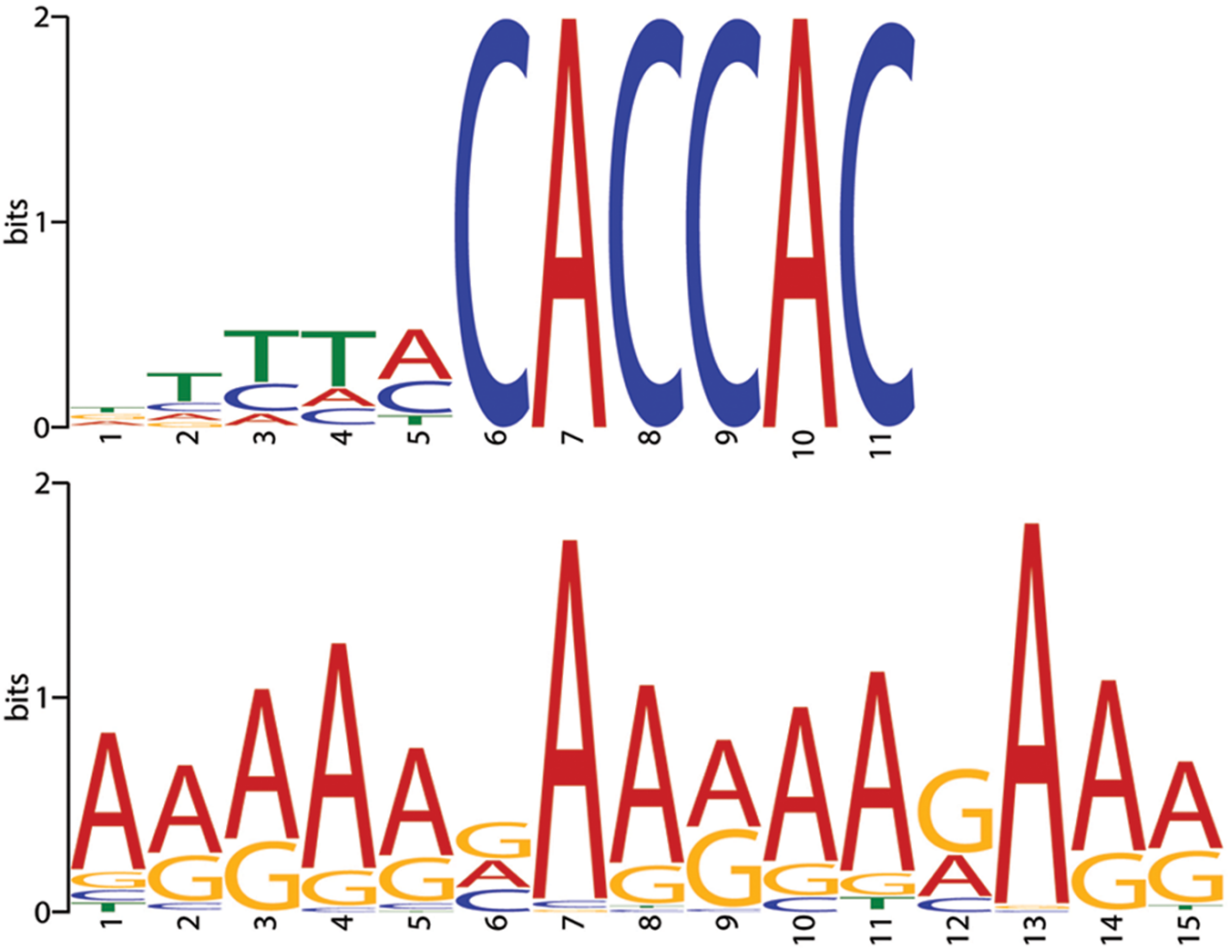
Sequence motifs associated with T-DNA–genome junctions sites. The motifs CACCAC (P-value = 1e-147, HOMER, E-value = 1.7e-234, MEME) and A-rich (P-value < 1e-21, HOMER, E-value = 2.3e-483, MEME) were associated with T-DNA–genome junction sites.

Some sequence motifs were found to be significantly enriched at the T-DNA-genome junctions (Fig 3). The most significant motif found to be enriched by both tools (P-value = 1e-147, HOMER, E-value = 1.7e-234, MEME) is a motif whose consensus, CACCAC, matches to the left border of the T-DNA (Fig 3). This motif is present in 25 percent of the input sequences (585 bins). The motif can be indicative of microhomology-mediated integrations that are frequently observed during non-homologous end-joining events [33] and during T-DNA integration [4,5,34]. Other motifs significantly enriched at junctions (P-value < 1e-21, HOMER, E-value = 2.3e-483, MEME) tend to be AT-rich (Fig 3).

As a control, all three restriction enzyme consensus motifs, GAATTC of EcoRI (P-value = 1e-119), TCTAGA of XbaI (P-value = 1e-112), and AAGCTT of HindIII (P-value = 1e-46), were found by HOMER in the motif analysis of the bins (S3 Fig). This is important because the presence of one of these restriction sites is expected to be associated with each detected junction based on the mechanism underlying the adapter ligation-mediate PCR method.

### Contrasting epigenetic biases between selected and unselected junctions

**Fig 4:**
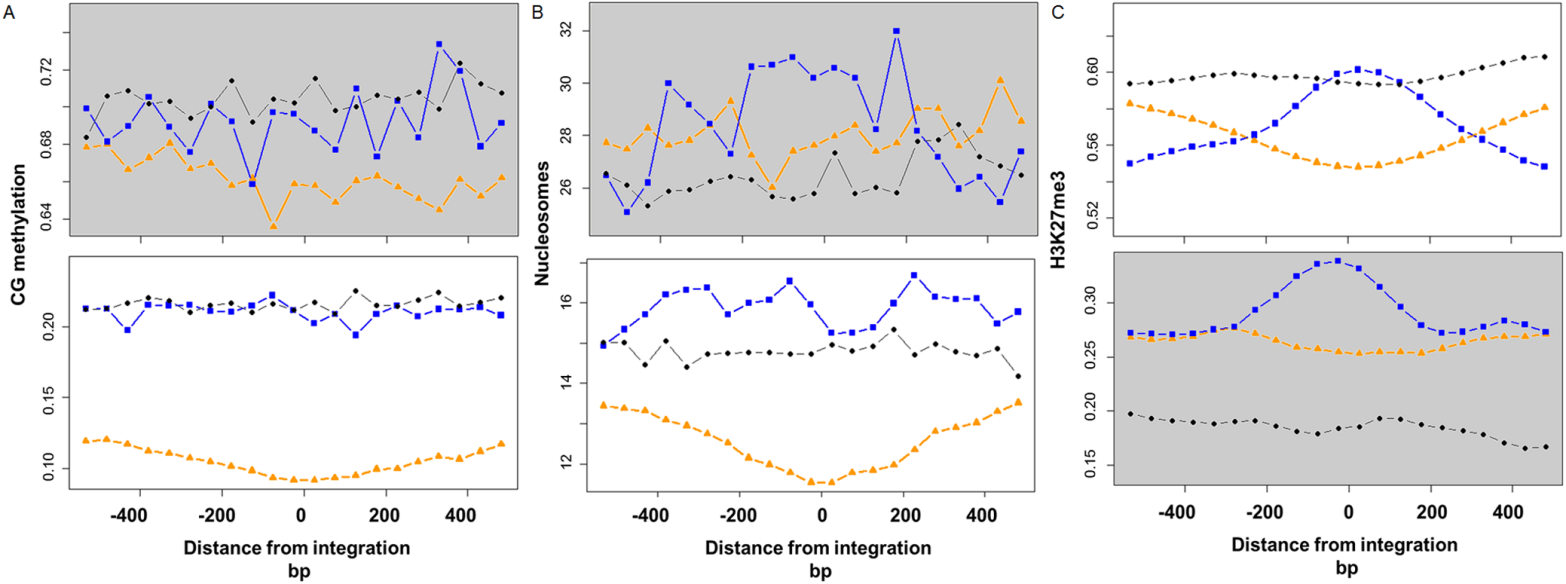
Epigenetic modifications around T-DNA–genome junctions. The analysis was performed separately in pericentric (grey background) and in remaining (distal) chromosomal regions. The up and downstream regions to T-DNA–genome junctions (represented as the 0 bp) are shown on the X axis. The Y axis represents the arbitrary level of the epigenetic markers. Blue squares–unselected T-DNA–genome junctions. Orange triangle–integrations under selective conditions [13]. Black circles–control, random genomic positions.–. A- CG methylation. B–Nucleosome occupancy. C- H3K27me3 modification.

Epigenetic modifications are known to be involved in DNA recombination events [21,22,35,36]. We performed a detailed investigation of epigenetic marks around T-DNA-genome junctions with or without selection. Since both centromeric and pericentric regions have distinct epigenetic character relative to the rest of the genome, we split the analysis for centromeric/pericentric and distal genome regions. We looked at the epigenetic landscape 500bp up and downstream from each junction. We found significant epigenetic differences between selected and unselected events. In distal regions, DNA methylation at sites of unselected junctions showed patterns similar to random, and that were significantly different (p = 9.21E-170, u-test) from the selected junctions which showed almost no methylation at the site of integration (Fig 4A). In pericentromeric regions, methylation patterns were close to random and differences between selected and unselected junctions were not significant (p = 0.4, u-test) (Fig 4A).

Selected events showed very low nucleosome occupancy, especially at integration sites (Fig 4B). By contrast, in distal regions, unselected events showed higher nucleosome occupancy than selected events (p = 1.35E-188) and slightly higher occupancy than a random dataset (p = 9.97E-27) (Fig 4B). A similar trend was also observed in pericentric regions (p = 1E-04, compared to selected events; p = 1.12E-06, compared to control) (Fig 4B).

In distal regions, we found a peak for H3K27me3 around the T-DNA-genome junctions of unselected events (p<0.0035, compared to random control), while there is a “valley” for the selected integration events (Fig 4C). In pericentric regions, H3K27me3 levels around T-DNA-genome junctions were overall lower than in distal regions, with higher level in selected compared to unselected regions (Fig 4C).

## Discussion

We performed an analysis of the genetic and epigenetic landscape of T-DNA-genome junctions in the *Arabidopsis* genome. We performed a high-throughput analysis of unselected T-DNA-genome junctions from Agrobacterium-infected *Arabidopsis* roots and we also reanalyzed selected T-DNA integrations [13] using recent data on the epigenome [17,22]. The T-DNA-genome junctions that we isolated are most likely integration sites because they were amplified from high molecular weight DNA and they contained hallmarks of T-DNA integration, such as, microhomologies between the T-DNA and the integration site [37]. Moreover, only a small number of events were found at time 0, compared to later time points (S1 Fig), and we used several filtering criteria in order to eliminate junctions formed from primers mis-annealing to the genomic sequences as described in the results. Nevertheless, because we focused on the left border junctions, it is possible that some of the events are abortive integration events, or represent intermediates in the integration process and are not yet joined with genomic sequence at the right border. Our choice to focus on the left border was due to several reasons: (i) the protocol from which we derived our adapter ligation-mediate technique on [27] was designed to recover left but not right borders by PCR [38]; (ii) attempts for right border amplification yielded a lower success rate relative to left border junctions [14], and this could be misleading when trying to compare integration kinetics at both borders; (iii) many right borders form concatamers with other T-DNA molecules [39]. These concatamers (which also form extrachromosomally [40]) would create a stretch of end-to-end T-DNA sequence that could be amplified by the right border primer alone, and this could result in biasing in favor of the amplification of end-to-end T-DNAs and obscure the identification of true junctions between the right border and the host genome.

### Timing

The approach used here enabled the detection of T-DNA junctions independently of selection or expression of markers. Junctions were found as early as 6 hours after root infection. Earlier studies that were performed without selection detected mRNA expression from a promoter-less GUS transgene that suggested integrations occur as early as 18 h post infection in BY2 cells [41]. Since mRNA transcription initiation, elongation, and accumulation to a detectable amount of transcript can delay the detection of the integration of a reporter gene, it is very possible that initial junction formation took place considerably earlier than 18 hours. Our results provide new and direct evidence for T-DNA–genome junction formation in *Arabidopsis* roots as early as 6 hours post-inoculation. Further work is needed to determine the exact timing of the very first integration events. An interesting finding with relevance to the kinetics of integration is that we observed a reduction in the number of integration intermediates between T6 to T24. This may reflect true kinetics of integration, such as stress response (e.g. plant defense) limiting later integrations, or it might be that the high level of bacteria used in our experiment caused apoptosis of root cells soon after infection.

### Sequence biases at T-DNA–genome junctions

On the basis of a large sample of T-DNA–genome junctions, we found the junction landscape to be unbiased toward genes, promoters or other tested genomic features. In fact, we even detected a small but significant bias toward intergenic regions (Fig 2B). At the sequence level, we found microhomology to the T-DNA left border to be involved in at least 25% of the junctions. This is probably an underestimate as we detected the CACCAC motif from the left border but we did not consider very short (1–2 bp) nucleotide identity in the analysis. It may be that the microhomology detected at the junctions is the result of a microhomology-mediated end joining process. We also found that unselected junctions are enriched with AT-rich motifs similar to the motif associated with meiotic recombination [22]. It may be that this sequence bias reflects the occurrence of DNA DSBs that serve as entry points for T-DNA integration, similar to the DSB events that precede meiotic recombination.

### Epigenetic biases

The epigenetic landscape of unselected T-DNA–genome junctions differed significantly from selected integrations. T-DNA integrations under selection were located in “open chromatin” regions with very low cytosine methylation, low nucleosome occupancy, and low H3K27me3. By contrast, without selection the T-DNA–genome junctions showed a bias towards regions with marks of heterochromatin such as high nucleosome occupancy and H3K27me3 (in particular in pericentric regions) but not of high cytosine methylation (Fig 4). Interestingly, Hi-C studies of chromatin packing have shown that such epigenetic marks (high H3K27me3 and high histone occupancy) define a chromatin state of small heterochromatin regions embedded in euchromatin that are “sticky”, highly interacting regions [19,24–26]. We speculate that such structures might serve as “landing” sites for incoming T-DNA—a mechanism that may protect the genome through capturing and silencing of incoming DNA into regions prone to breaks or nicks [24]. It is also possible that the T-DNA-VirE2 or VirD2 complex interacts with host chromatin factors [42,43] that drive it to heterochromatin regions.

Our results extend an earlier study that showed the difference between selected and unselected events [14]. However, this earlier study, based on a small number of events (n = 117), reported on randomness of integration while we show a clear bias for specific genetic and epigenetic markers. What is the cause for the different integration patterns between selected and unselected events? The association of selection based integration with open chromatin is most likely due to the need of the selection marker to be expressed in order for the transformed plant to survive. It is thus reasonable to assume that the biases seen with selected events do not reflect an integration bias but rather the silencing of the transformation marker. While alternative hypotheses are possible, there is a strong biological basis supporting silencing as an explanation to the observed bias between selected and unselected events [3,44–46]. This work, which provides a genome-wide analysis of genetic and epigenetic patterns of selected and unselected T-DNA–genome junctions, contributes to a better understanding of the process of T-DNA integration. It opens new prospects to study how the interaction between the incoming DNA and the chromatin structures determine patterns of integration, and it argues against the widespread notion that the heterochromatin is not an accessible region.

## Materials and methods

### Plants

Wild-type *A*. *thaliana*, Columbia-0 ecotype, seeds were surface sterilized in a solution of 30% bleach and 0.1% Triton X-100 for 10 minutes in a 50 ml conical vial (inverting every 2–3 minutes). The seeds were rinsed at least 3x in ~20 ml of sterile water. A P1000 pipette was used to transfer ~100–150 seeds onto square plates containing Gamborg’s B5 media (1.8% agarose; 20 g/L sucrose). Seeds were dispersed in a line ~3 cm from one edge of the plate. Plates were sealed with Parafilm and the seeds vernalized by placing in the dark at 4°C for 48–72 hours. Plates were removed from 4°C and placed in a growth chamber at 22°C with constant light in an upright position so that the roots grow down along the surface of the agar. The seedlings were allowed to grow for 10–12 days before infecting.

### Bacteria

A frozen stock of *Agrobacteria tumefaciens* strain At1529 (GUS with intron-containing T-DNA binary vector pBISN1 in strain A348; Narasimhulu *et al*. 1996 [47]) was freshly streaked onto a YEB plate containing 50 μg/ml kanamycin and 10 μg/ml rifampicin and grown at 28°C for 48 hours. A 5 ml culture of YEB media (25 μg/ml kanamycin, 10 μg/ml rifampicin) was inoculated from a single *A*. *tumefaciens* colony and grown overnight with shaking at 28°C. The next morning the overnight culture was diluted 1:20 into fresh media and grown as above until an OD_600_ of ~0.8 was reached. The bacteria were pelleted at 9,000xg for 5 min, the supernatant removed and the cells resuspended in 0.9% NaCl solution. This pelleting and rinse was repeated one more time and the cells were diluted down to 1:100 in 0.9% NaCl.

### Arabidopsis root infection

Under sterile conditions the roots of the ~11 day-old seedlings were cut into 2–3 mm segments with a scalpel. Using sterile tweezers, root segments were collected into bundles of 50–100 and placed on an MS plate (with 10 g/L sucrose). Root tips were not included in the bundles. Once the bundles were prepared they were inoculated with the freshly rinsed *A*. *tumefaciens* bacteria, enough bacterial culture was added to cover the root bundles entirely. After 15 minutes the excess bacteria and liquid were removed gently with a pipette. Root bundles for the 0-time point were transferred to a microcentrifuge tube, rinsed 3x in 0.9% NaCl and quickly frozen in liquid nitrogen. The plates containing the remaining root bundles were sealed in Parafilm and incubated at 22°C in the dark. At 6 hours and 24 hours post-infection root bundles were removed from the plate, transferred to a microcentrifuge tube and washed 3x in 0.9% NaCl, frozen in liquid nitrogen and stored at -80°C. At 48 hours post-infection one or two root bundles were stained for GUS expression as described in Zhu *et al*., 2003 [48]. In most cases 70–90% of cut root ends in a bundle were positive for GUS staining. If the percentage of GUS-positive root segments was lower than 70% the experiment was terminated because of the low transformation efficiency.

### Arabidopsis DNA extraction

Root tissue from each time point was processed in parallel and care was taken to keep samples separate from one another in order to reduce cross contamination. The lyophilized root bundles were physically disrupted using a Qiagen TissueLyzer set at 30 Hz for 2 min with 3 mm tungsten-carbide beads. Total genomic DNA was isolated from the root tissue using the Sigma Gene-Elute kit (G2N70-1KT) according to the manufacturer’s protocol. The 200 μl eluted genomic DNA was ethanol precipitated using 100 μl of 7.5 M ammonium acetate and 600 μl of ice cold >95% ethanol and spinning for 20 min at 0°C. The DNA pellet was washed once with 70% ethanol, air-dried for ~10 min, resuspended in 10 μl of TE buffer and stored at 4°C. To isolate the high molecular weight DNA from lower weight DNA that may contain unintegrated T-DNA, the total DNA was loaded onto a 0.7% agarose gel and run at 90 volts for 1 hour. The band containing the high weight DNA was excised with a scalpel and the DNA extracted from the gel fragment on glasswool treated with Sigmacote in a nested microcentrifuge tube column. The DNA-containing liquid extracted from the column was ethanol precipitated as described above and resuspended in 20 μl of TE buffer. The DNA was stored at 4°C for short-term storage (<48 hours) and -80°C for longer storage.

### Adapter ligation-mediated PCR

The adapter ligation-mediated PCR is based on the protocol described by O’Malley *et al*., 2007 [27]. Their published protocol was developed to identify T-DNA junctions in stably transformed, clonal *Arabidopsis* plants using Sanger sequencing methods to identify and map the T-DNA-flanking regions to the *Arabidopsis* genome. We adapted their protocol to identify T-DNA-flanking regions from a population of *Arabidopsis* root cells containing potentially thousands of independent T-DNA–genome junctions spread throughout the *Arabidopsis* genome. Additionally, instead of generating a clone library from a PCR amplicon and sequencing independent inserts, we used the Illumina HiSeq2000 sequencer platform to sequence the amplicons directly after the adapter ligation-mediated PCR. The O’Malley et al., 2007 protocol was followed with some modifications as described here. The primers used to generate the adapters and for subsequent PCR are shown in Table 1 Adapters with specific overhangs for EcoRI, HindIII or XbaI were generated by annealing the long and short adapters as follows: mix 20 μl of 5 μM long strand with 20 μl of 5 μM short strand adapters for either EcoRI, HindIII or XbaI adapters in 1,210 μl of 1 mM Tris, pH 8.3, in a microcentrifuge tube. Vortex the tubes and place them in a waterbath at 96°C for 2 min and allow to cool to room temperature over 30 minutes.

**Table 1:** 

NAME	SEQUENCE
Long Strand1	GTAATACGACTCACTATAGGGCACGCGTGGTCGACGGCCCGGGCTGC
Short HindIII	[PHOS]AGCTGCAGCCCG[AmC7-Q]
Short EcoRI	[PHOS]AATTGCAGCCCG[AmC7-Q]
Short XbaI	[PHOS]CTAGGCAGCCCG[AmC7-Q]
TDNALeftLBa1	TGGTTCACGTAGTGGGCCATCG
LBminus11	GTCTAAGCGTCAATTTGTTTACACCAC
-70 primer + TIME BARCODE(X)	XXXXXX GGTGAAAAGAAAAACCACCCCAGTAC
-30 primer + TIME BARCODE(X)	XXXXXX GTACATTAAAAACGTCCGCAATGTGTTATTAAG
-11 primer + TIME BARCODE(X)	XXXXXX GTCTAAGCGTCAATTTGTTTACACCAC
BARCODE(X) 0 MINUTES	PuPyPuPyPuPy (RYRYRY)
BARCODE(X) 6 HOURS	PuPuPyPyPuPu (RRYYRR)
BARCODE(X) 24 HOURS	PuPyPyPuPyPy (RYYRYY)

Pu / R = Purine; Py/Y = Pyrimidine

For each time point, high molecular weight DNA from three or four independent root infections was pooled to create a source of template DNA for adapter ligation-mediated PCR. Each root infection time point used 4–5 bundles of roots, with each bundle containing up to 100 root segments from dozens of *Arabidopsis* seedlings, so that template DNA from each time point contained potentially thousands of independent T-DNA–genome junctions. From the pooled high molecular weight DNA, for each time point separately, 30–50 ng of DNA were subjected to a combined digestion and ligation process. The DNA was incubated with 2 units each of EcoRI, HindIII and, XbaI, along with 10 units of T4 DNA ligase, 0.25 μl of each adapter in NEB ligation buffer in a 40 μl volume. The combined digestion-ligation reaction was allowed to incubate at room temperature overnight. The following day the first-round PCR reaction was set-up using 8 μl of the digestion-ligation product as a template in a thin-walled 200 μl PCR tube. In addition to the template DNA the 20 μl PCR reactions contained 2 μl of 10X PCR buffer, 0.8 μl of 10 mM dNTPs, 1 μl of the LBa1 primer, 1 μl of the AP1 adapter primer, 0.1 μl Taq polymerase (either Sigma Taq or Takara) and 7.1 μl of PCR grade water. The tubes were placed in the thermal cycler and run for 10 cycles at 98°C for 20 seconds, 72°C for 2.2 minutes, then for 15 cycles at 96°C for 20 seconds, 67°C for 2.2 minutes. For the second-round of PCR, 0.5 μl of the first round reaction was used as a template with the same set-up as before, but instead using 1 μl of the nested LB primer and 1 μl of the nested AP2 adapter primer. The tubes were placed in a thermal cycler and run first for five cycles at 96°C for 20 seconds, 88°C for 20 seconds, and 72°C for 45 seconds, and then for 23 cycles at 96°C for 20 seconds, 62°C for 20 seconds and 72° C for 45 seconds. Because we intended to sequence the PCR amplicons and not generate clone libraries we made a number of changes to the nested LB primers. We used three LB primers with 3’ terminal base at positions, -11, -30 and -70, respectively, relative to the canonical LB T-DNA cleavage site. Each of these three versions of the LB primer were generated with different barcodes, one specific for each time point, that would allow us to pool reactions for Illumina sequencing (see Table 1). The decision to use a set of three LB primers at increasing distance from the canonical cleavage site is because the LB is often imperfectly processed, resulting in deletions that can exceed 100 bp. However, with the Illumina short read sequencing technology we could not use a single LB primer set well back from the LB cleavage site because in many cases sequencing from the LB in the absence of any LB deletion would not allow us to sequence far enough into the adjacent *Arabidopsis* DNA to map the location in the genome. By using three separate primers at varying distances from the LB cleavage site we increase our odds of identifying junctions that occur with the expected LB processing and also those that might be formed in cases where up to ~65 bp of the LB is removed in the T-DNA integration process. In the case of both the round-one and round-two PCR reactions, 5 μl were loaded onto a 1% agarose gel and run to check for amplification. In all cases, no product is visible in the first-round reactions, but in the second-round reactions a smear of DNA amplicons can be seen extending from ~50 bp to 500 bp. This smear is expected because we are amplifying thousands of independent T-DNAs junctions and each junction can vary in size. Two or three independent round-two PCR reactions were run and the products from each time point combined and the concentration determined using a NanoDrop 1000 instrument. Equal amounts of DNA from each time point of the second round PCR products were pooled and then cleaned using a Qiagen MinElute kit. The pooled amplicons were quantified by NanoDrop and 30 μl of ~10ng/μl of DNA was used for Illumina sequencing.

### Illumina sequencing

Sequencing was performed in the INCPM center at the Weizmann Institute using the TruSeq ChIP Library Preparation Kits and HiSeq2000 sequencer. Read length was 100bp.

### Data filtering and alignment

To ensure the use of high quality reads, raw reads from the Illumina sequencer were filtered using PRINSEQ [49]. Only reads with Phred score above 25 for every base were used for the downstream analysis. Reads that matched T0 reads were removed from the other time points (T6, T24) using CD-HIT [50]. Out of the remaining reads only reads containing at least one of the primers used from within the T-DNA LB, plus 4 bases downstream of the primer, were used for the analysis. The reads remaining after filtering were mapped to TAIR10 Arabidopsis genome using command line blast [51] (version blastall 2.2.26 with the blast program—blastn). Each read was required to contain at least 22 bp at the 3’ end of the read that match to the Arabidopsis genome. The position of the best alignment was chosen for every read. Namely, if a read mapped to a unique place over the genome this place was chosen. In case of multiple possible places, the read with the best bit score was chosen. Positions that mapped +/- 10 from each other and originate from the same primer were collapsed to one. Finally, T0 reads were mapped to the genome and divided to bins of 400bp each, locations which showed preference to T0 positions, higher than mean plus two times the standard deviation, were excluded from the analysis.

### Data analysis

**Sequence motif analysis.** The genome was divided into non-overlapping bins of 400bp. Bins that contained at least one T-DNA–genome junction were used for the analysis of the unselected dataset. Selected junctions were taken from TAIR10 annotation files based on publicly available data [13]. Motif analysis was done using MEME [32] and HOMER [30]. In HOMER the junction dataset was tested versus the whole genome.

**Association with genomic features and epigenetic marks.** TAIR10 genomic features were used for the genomic features analysis. Epigenetic data were provided by Assaf Zemach and were described in [17]. The epigenetic data were binned every 50 bp. The mean values 500 bp up- and downstream of each of the sites were calculated for all the occurrences in a certain region.

## Supporting information

S1 Fig T-DNA–genome junction dynamics. Bars show the number of raw reads produced from each time point 0 hours (control–T0), 6 hours (T6) and 24 hours (T24) post-infection.
(TIF)

S2 Fig Association of genomic features with selected (orange) and unselected T-DNA–genome junctions (blue). The genomic distribution of T-DNA–genome junctions across Chr 1–3, 5 (Chr 4 is in Fig 2). Unselected T-DNA–genome junctions (circles, and smoothed blue line) do not show correlation with the distribution of TE (red line) and promoters (green line) while T-DNA integrations under selective conditions correlate with promoters.
(TIF)

S3 Fig Sequence motifs found by HOMER in the motif analysis match to all three restriction enzymes used in the experiment, GAATTC of EcoRI (P-value = 1e-119), TCTAGA of XbaI (P-value = 1e-112), and AAGCTT of HindIII (P-value = 1e-46). (TIF)
