# Supplementary figures and images for: T-DNA-genome junctions form early after infection and are influenced by the chromatin state of the host genome
Source: PLoS Genet. 2017 Jul 24;13(7):e1006875. doi: 10.1371/journal.pgen.1006875 (PMC5546698; doi:10.1371/journal.pgen.1006875)

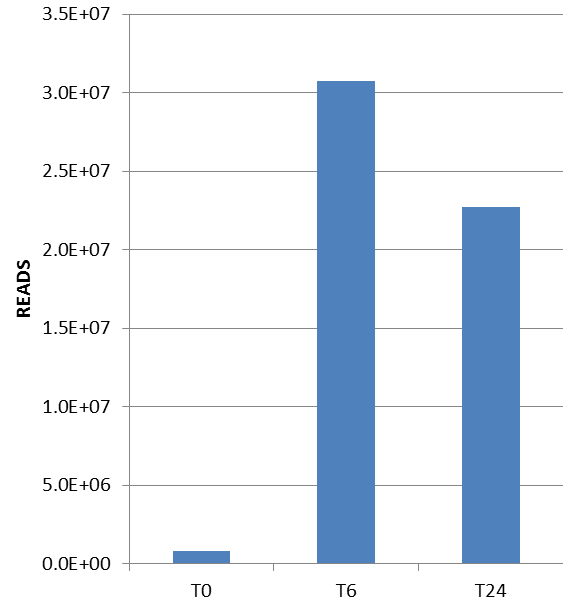

Supplement: S1 Fig — Bars show the number of raw reads produced from each time point 0 hours (control–T0), 6 hours (T6) and 24 hours (T24) post-infection. (TIF) [file pgen.1006875.s001.tif]

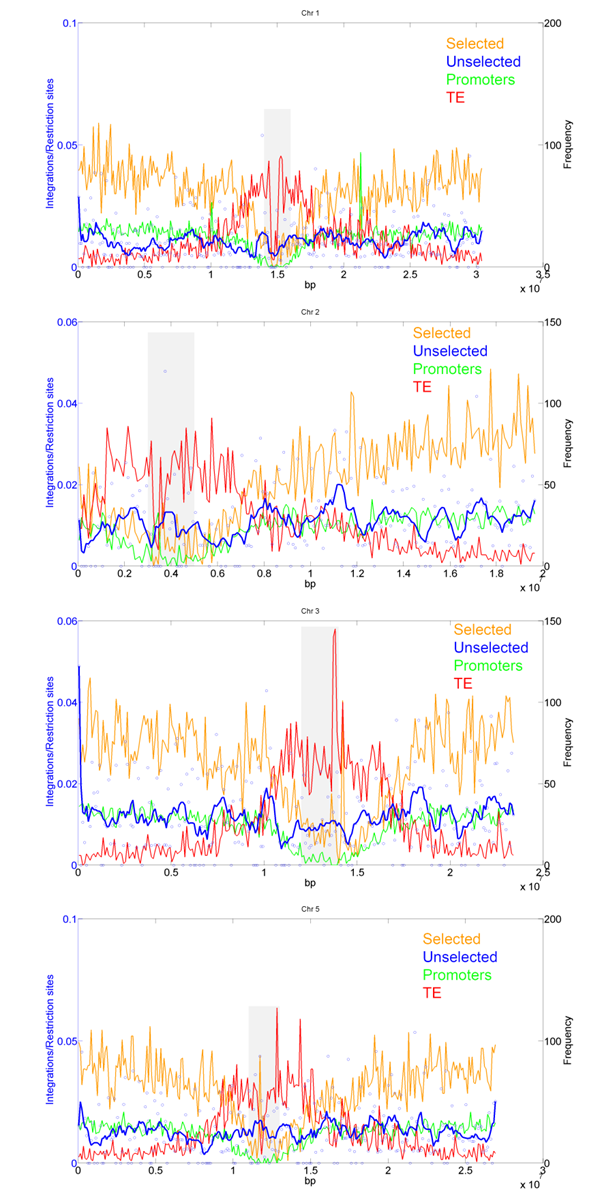

Supplement: S2 Fig — The genomic distribution of T-DNA–genome junctions across Chr 1–3, 5 (Chr 4 is in Fig 2). Unselected T-DNA–genome junctions (circles, and smoothed blue line) do not show correlation with the distribution of TE (red line) and promoters (green line) while T-DNA integrations under selective conditions correlate with promoters. (TIF) [file pgen.1006875.s002.tif]

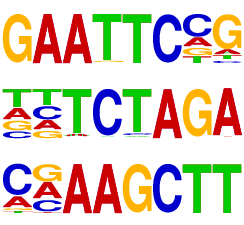

Supplement: S3 Fig — (TIF) [file pgen.1006875.s003.tif]
